# A plea of those who are affected most by HIV: The utterances by women who inject Nyaope residing in the City of Tshwane Municipality, Gauteng

**DOI:** 10.4102/phcfm.v13i1.2416

**Published:** 2021-08-16

**Authors:** Moganki H. Lefoka, Thinavhuyo R. Netangaheni

**Affiliations:** 1 Department of Sociology, Faculty of Human Sciences, University of South Africa, Pretoria, South Africa

**Keywords:** women who inject drugs, Nyaope, needle exchange programme, HIV, harm reduction

## Abstract

**Background:**

Nyaope injecting practice brought the field of Human Immunodeficiency Virus (HIV) prevention and Substance Use Disorder (SUD) together. It is complex and requires multidisciplinary approach. Women who use drugs face individual, social, and structural factors that fuel their vulnerability to contract HIV, and other blood-borne infections. Women Who Inject Drugs (WWID) are a subpopulation that is neglected from HIV prevention and SUD treatment interventions, and are hardly the subject of surveys. In order to fully address the HIV epidemic among WWID it is imperative that they become part of the process of finding solutions.

**Aim:**

This study explored the strategies to curb HIV incidence among Women Who Inject Nyaope (WWIN), residing in City of Tshwane Municipality, Gauteng Province.

**Setting:**

The research was conducted within COSUP. COSUP was considered more appropriate as it is a harm reduction based organisation.

**Methods:**

The study utilised the qualitative research approach. Semi structured interviews were conducted with 24 women with a history of injecting Nyaope aged between 19 to 35 years. The data was analysed using thematic data analysis.

**Results:**

Health intervention, economic intervention and educational intervention was stressed as key strategies to curb HIV among WWIN. Needle exchange programmes, condom distribution, PrEP, HIV Testing and Counselling, employment opportunities, support groups and awareness campaigns if implemented, can yield positive outcomes in curbing HIV among WWID.

**Conclusion:**

Mechanisms to curb HIV among WWIN exist, and when implemented, they have the potential to address high HIV incidence among women who inject Nyaope.

## Introduction

Nyaope injecting practice has brought the field of Human Immunodeficiency Virus (HIV) services and Substance Use Disorder (SUD) treatment together. Nyaope is a drug preferred by many drug users in townships, especially in Gauteng.^1^ It has been reported that the use of Nyaope has increased in recent years mainly amongst young African and mixed race males in South Africa (SA).^2^ Nyaope is a novel fashionable drug that is normally used in many townships in SA. The full make-up of Nyaope is not known, even though most researchers concur that heroin is the main component, with rumors of the inclusion of antiretroviral having been documented.^3^ Nyaope is called by different names in different areas. In Gauteng townships such as Soshanguve, Mamelodi and Soweto, it is called Nyaope, whilst in KwaZulu-Natal (KZN) townships such as Umlazi, Kwa-Mashu and Inanda, it is called Whoonga.^4^

The practice of Nyaope injecting needs to be researched and calls for an integrative approach. Substance Use Disorder treatment and HIV services for People Who Inject Drugs (PWID) in SA are limited and are primarily provided by civil society organisations.^5^ Publicly funded SUD treatment services are almost exclusively abstinence-based.^5^ Despite the upsurge in heroin use and an increase in injecting use, there is no established framework to deal with the public health crisis that predictably follows the increased dependent use of unregulated opioids.^6^

Nyaope is a multi-faceted challenge, and its addiction is caused by a variety of factors. Nyaope addiction needs a multi-faceted approach; it poses unique challenges in different communities. The challenges cannot be addressed without engaging the users to shed light on their challenges, coping resources, experiences, risks, needs and hopes.^7^

The Joint Nations Programme on Human Immunodeficiency and Acquired Immunodeficiency Syndrome (UNAIDS) has reported that globally, the statistics of PWID is about 16 million, of whom, 3 million are projected to be HIV infected,^8^ and further highlighted that the incidence of HIV infection globally for all ages has declined by 25% between 2010 and 2017, but HIV infections amongst PWID has increased.^9^ The incidence of HIV infection amongst PWID appears to have risen over the past years from 1.2% in 2011 to 1.4% in 2017.^9^ It is estimated that women account for 27% of all PWID in SA, and 19.4% of Women Who Inject Drugs (WWID) are HIV infected.^10^

Reducing HIV incidence amongst women who inject Nyaope can only be achieved through improving HIV prevention and treatment for PWID as an urgent international priority; as identified by several high-level initiatives, including the Global Fund to Fight Acquired Immunodeficiency Syndrome (AIDS), Tuberculosis (TB) and Malaria, and the UNAIDS 90–90–90 targets. These initiatives are aimed at substantially scaling up access to, and the effect of, HIV treatment by 2020.^11^

Women Who Inject Drugs are a subpopulation that is neglected from HIV prevention and SUD treatment interventions^12^ and is hardly the subject of surveys.^13,14^ To fully address the HIV epidemic amongst WWID, they must be part of the process of finding solutions.

Women Who Use Drugs (WWUD) face multiple individual, social and structural factors that fuel their vulnerability to HIV, and other blood-borne infections.^8^ Women Who Use Drugs, regardless of whether they are injecting or not, experience numerous issues that increase their vulnerability to HIV. These include concomitant sex work, sexual transmitted infections (STIs), viral hepatitis, mental health problems, reproductive health issues, stigma, child care, violence and lack of access to health services including HIV prevention, care and treatment.^8^ A study conducted in SA has highlighted that WWUD suffer human rights abuse at all levels.^15^ The effects of drug use are more severe in women than their male counterparts. Women experience problems, such as economic hardship, greater drug dependence, health risks, as well as involvement in HIV-related high-risk behaviour.^16^

Human Immunodeficiency Virus amongst PWID is unjustly higher compared to the general population, with PWID accounting 28 times higher prevalence.^13^ Globally, the statistics of PWID is about 16 million, of whom, 3 million are projected to be HIV-infected.^8^ It is estimated that women account for 27% of all PWID in SA, and 19.4% of female Nyaope injectors are HIV infected.^10^ United Nations Office on Drugs and Crime (UNODC) reported that the sharing of injecting-equipment is one of the main drivers of HIV transmission amongst PWID.^15^ People Who Inject Drugs are also at risk of HIV transmission through unprotected sex, which is predisposed by links to sex work, transactional sex and drug-related effects. Several social and structural factors contribute to the increased HIV burden amongst PWID.^5^

Women Who Inject Drugs are more likely to participate in drug, and sexual risks than males who inject drugs due to severe discrimination, and cultural stigma perpetuated on them.^12^ Women Who Inject Drugs tend to be dependent on drugs faster than their male counterparts, inject more frequently per day, have intimate partners who inject, contract and die from HIV/AIDS, and have severe combined risks. This is partly because most of them engage in sex work to raise money for drugs.^17^ Azim et al.^8^ further argue that WWUD engage in unprotected sex with their clients, as well as their intimate partners, and have higher rates of STI. They are also at risk of experiencing sexual violence and incarceration.

There is a lack of programmes that integrate SUD and HIV services for PWID in SA.^5^ Regardless of the acknowledgement of the effects of drug use on HIV transmission, there are inadequate HIV harm reduction services in SA. There is only one harm reduction programme that is funded by the South African government within the whole of SA. Community Oriented Substance Use Programme (COSUP), which is implemented by the University of Pretoria, Department of Family Medicine, and funded by the City of Tshwane Municipality (CoT), is the first publicly funded community-based programmatic response to the use of unregulated drugs in SA.^18^

Based on the connection between HIV risk behaviour and drug use, it can be justified that strengthening efforts to offer integrated HIV harm reduction and SUD treatment services should be prioritised. Although the drafting of SA’s Third National Drug Master Plan (2013–2017) gave eminence to the necessity of addressing drug abuse as part of comprehensive HIV prevention efforts, programmes targeting people who use drugs, with a sole purpose of addressing drug use and sexual risk behaviour in SA, are scarce.^19^ South Africa’s first needle and syringe programme started in 2014 in Cape Town, extending to Durban and Pretoria in 2015.

The study aimed to investigate strategies to mitigate or curb HIV incidence amongst women who inject Nyaope residing in CoT, Gauteng.

## Methods

### Study design

Qualitative methods were used to explore and describe strategies that could be used to curb or mitigate HIV incidence amongst women who inject Nyaope.

### Setting

The research was conducted within COSUP sites across CoT. By mid-2019 there were 17 functional and viable sites available to clients in CoT. Twelve included drop-in centres that provided access to ablution facilities, nutrition, computers, other psychosocial services and safe spaces to socialise.^18^ Community Oriented Substance Use Progamme was considered more suitable as it is a harm reduction-based organisation that offers Opioid Substitute Therapy (OST) and Needle Exchange Programme (NSP) across CoT.

### Study population and sampling strategy

The population in this study were women, between the ages of 18 years and above who reside in the CoT Municipality with a history of injecting Nyaope for more than 6 months. The participants were accessing SUD services at COSUP sites across CoT. The researcher sampled 24 participants from the population. Fifteen who are currently injecting Nyaope and nine with a history of injecting Nyaope were interviewed. Inclusion and exclusion criteria were followed during the sampling process.

Purposive sampling method was used to recruit participants from COSUP’s NSP and OST social work caseloads. All females accessing services at COSUP with a history of injecting Nyaope, who meet inclusion criteria were invited through the aid of site social workers to participate in the study.

Six months was considered adequate to exclude participants who might not have enough living experiences as Nyaope injectors as the researcher was interested in participants with rich experience as Nyaope injectors. The researcher understood that female Nyaope injectors are a hidden population and if 1 year plus of injecting experience is required, the researcher might struggle to find suitable participants.

The site social workers in preparation of data collection handed over the participants’ consent forms and assisted those who could not understand English with understanding the content of the forms. The consent forms were written in easy English, free of vocabularies, jargons and legalese so that the participants can comprehend what was written before they consent to participate. The researcher also checked with the participants if they understood the purpose of the research before collecting data. The risks, benefits and their rights were discussed at length with the participants in the presence of a social worker before they consented to participate in the study. All participants were allowed an opportunity to accept or decline to participate in the research study. No one was forced or lured with money to participate. One participant did not want to participate and the researcher respected the decision.

**TABLE 1:** Inclusion criteria.

Recovered Nyaope injectors	Current Nyaope injectors
The participant must be a female with 6 months or more of injecting Nyaope before stopping	The participants must be a female with 6 months or more history of injecting Nyaope
The participant must reside in the City of Tshwane Municipality	The participant must reside in the City of Tshwane Municipality
The participant must be 18 years and above	The participant must be 18 years and above

**TABLE 2:** Exclusion criteria.

Recovered Nyaope injectors	Current Nyaope injectors
Participants who have relapsed	Participants who are intoxicated
Participants who are intoxicated	Participants who are not linked to an organisation
Participants who are not linked to an organisation	

### Data collection

After permission to collect data was granted by COSUP Management. The researcher liaised with the site social workers for data collection. The researcher collected data from 24 participants using semi-structured interviews. All the interviews were audio-recorded, and the interviews were kept safe to protect the information and maintain confidentiality. The researcher initially envisaged to interview 30 participants, 15 recovered injectors, and 15 current injectors, however, 15 current injectors, and nine recovered injectors were interviewed. The researcher did not interview 15 recovered injectors because of data saturation. The research reached data saturation after the researcher interviewed 24 research participants and then the researcher stopped. Saturation was reached after the 19th interview, but the researcher continued until the 24th interview. The researcher wanted to make certain that the interview process was not stopped prematurely, as a result, five more interviews were conducted after data saturation.

The researcher interviewed the participants at an environment they were familiar with, and in the presence of their social worker. This was done to assist the participant be at ease as the researcher was a male. The researcher gave the participants an option to choose a language suitable for them. Six participants were interviewed in English, and 18 were interviewed in Setswana. The researcher used qualitative observation.

### Data analysis

Data were analysed thematically using six phases as identified by Braun and Clarks.^20^

#### Phase 1: The researcher acquainted himself with the data

The researcher transcribed the audio recording from the 24 interviews verbatim. Through the transcribing process, the researcher had a chance to familiarise themselves with the content of the transcripts.

#### Phase 2: The researcher generated initial codes

The researcher read through the transcript one by one critically identifying initial codes. This allowed the researcher to code many codes that had the potential to form different themes.

#### Phase 3: The researcher searched for themes

The researcher started to organising codes linked to each other into potential themes. The researcher used a table form to organise the codes and themes orderly.

#### Phase 4: The researcher reviewed themes

The researcher reviewed all the themes. Sub-themes were created to accommodate themes, which are in alignment.

#### Phase 5: The researcher defined and named the themes; and

This phase started when the researcher was satisfied with the themes and sub-themes identified. It was identified what each theme is about and what aspect of data each theme captures.

#### Phase 6: The researcher produced the report

The researcher started writing up the thematic analysis of the data. The researcher strived to provide a concise, coherent, logical, non-repetitive and interesting account on the strategies of curbing or mitigating HIV incidence as reported by women who inject Nyaope.

### Ethical considerations

This article is part of a MA dissertation ‘Exploring the experiences of female Nyaope injectors residing in the CoT, Gauteng’. It was approved by the Research Ethics Committee of the College of Human Science, University of South Africa, with reference number 2019-CHS-0246.

## Results

### Biographical characteristics of the participants

Participants were between the ages of 22 and 35 years old. Fifteen participants were current injectors and nine were recovered injectors.

### Key findings

The key question in this study was: What are the strategies to mitigate or curb HIV incidence amongst women who inject Nyaope residing in CoT, Gauteng?

Three main themes and seven sub-themes emerged from the data analysis.

### Health intervention

The participants identified NSP, condom distribution, accessibility to Pre-exposure prophylaxis (PrEP) and HIV testing and counselling as health interventions that can be used to curb HIV amongst female Nyaope injectors.

### Needle and exchange programme

Participants acknowledge the value of NSP in their lives. They view NSP as an important and key programme to address the lack of accessibility to needles. The following narrative statements support the augment:

‘The service of providing injections is a good programme, it must continue.’ (Pt10, 32 years, black person)

‘The idea of coming here to give us needles, I think it was a brilliant idea.’ (Pt18, 32 years, black person)

‘NSP is important for the people that are injecting drugs. I think the NSP is very important and now that COSUP is offering it, it is something good because some people really struggle to get needles.’ (Pt13, 28 years, mixed race person)

**TABLE 3:** Themes and sub-themes.

Themes	Sub-themes
Health intervention	Needle exchange programme (NSP)
	Condom distribution
	Pre-exposure prophylaxis (PrEP)
	HIV Counselling and Testing (HCT)
Economic intervention	Employment opportunities
Educational intervention	Support groups
	Awareness campaigns

Participants, further report that the NSP programme is effective in addressing the spread of HIV amongst women who inject Nyaope. Participants acknowledge sharing needles contribute to the likelihood of contracting health conditions. This is supported by the following narratives:

‘Actually, to prevent the spread of HIV I would suggest giving them (WWID) needles.’ (Pt1, 23 years, black person)

‘What I would like to say is that they must not use these needles together (should not share needles) because many of my friends passed on using needles together (sharing needles) and did not know if the person is HIV (positive) and so on.’ (Pt12, 32 years, mixed race person)

Participants empathised that NSPs should be accessible to people who need it the most. Instead of providing the programme in offices, it should be available where women who inject Nyaope are situated, and enough needles should be distributed to WWID. The NSP service providers should adopt the same strategies condom distributors are utilising to address the accessibility of condoms. Participants reported that:

‘What I think we really need is the accessibility of injections and quantity, because most of us do not use the injections the same way. Some use less, others use more.’ (Pt3, 34 years, black person)

‘I would suggest that the needles should be distributed to them. The same way they distribute condoms, they should give needles the same way.’ (Pt19, 30 years, black person)

‘People (female Nyaope injectors) cannot always go there at the place where the needle exchange is, then they (NSP service providers) should go to more places.’ (Pt2, 32 years, mixed race person)

Some participants feel that to entirely cut the likelihood of contracting HIV amongst women who inject Nyaope, needles should not be available for use. They indicate that as long injecting practice exists, the likelihood of contracting HIV will never be eliminated. They empathise that once needles are out of stock or not available, drug injectors are bound to share needles, which expose the drug injectors to contracting HIV:

‘Distribution of needles should be cancelled, how you are going to protect us because you will give us injection as you give us in exchange programmes, at times they are out of stock, when they are out of stock, then you ask from someone else then at that time the person is sick.’ (Pt5, 31 years, black person)

### Condom distribution

Some participants acknowledge the self-determination of women who inject Nyaope to choose to participate in sex work or transactional sex. Risks linked with sex work and transactional sex can be lowered or reduced by the introduction and correct use of condoms during sexual intercourse. Condom distribution should be intensified, and female Nyaope injectors should have access to condoms. The distribution should reach women who inject Nyaope where they are to mitigate the risk of contracting HIV through sexual intercourse. As much as condoms can be distributed, women who inject Nyaope should be encouraged to use a condom consistently. Participants reported that:

‘You must distribute condoms to them, and encourage condom use.’ (Pt24, 29 years, black person)

‘Those who hustle (make money) by going to Rooival (informal brothel), at least they should have condoms.’ (Pt16, 31 years, black person)

‘Some of them are sex workers, encourage them to use protection at all times.’ (Pt1, 23 years, black person)

‘Let them be advised to always use condoms for safety. I cannot say let them be stopped because that is how they are able to get money to buy drugs. All in all, let them be advised to always have condoms with them in order to prevent themselves from getting sickness.’ (Pt9, 31 years, black person)

Condom distributors should not assume that everyone knows how to use condom accurately. Women who inject Nyaope should continuously be trained on condom use. A participant reported that male and female condoms should be distributed so that during sexual intercourse, a male will use their condom and a female will use her condom, this statement reflects a knowledge deficit on condom use and it highlights the need to continue educating people on condom use:

‘Let them be given male and female condoms that when they sleep together a male will wear one and female will wear hers’ (Pt23, 31 years, black person)

### Pre-exposure prophylaxis

Participants appreciate the value of PrEP medication as having the potential to curb HIV incidence amongst women who inject if it is more accessible to them. It is reflected in the narratives of the participants that there is a medical treatment available for people who are negative and have a higher risk of contracting HIV, but they lack knowledge of the treatment. One says it is a pill, while another says an injection:

‘Like, this injection should be available to everyone, for as long as you are negative, it should be available. The one that helps you not to be positive when you are negative at all even if you are being intimate. I do not know what they call it (referring to PrEP).’ (Pt23, 31 years, black person)

‘Is there no pill that they get, like, when a person is raped which they provide in hospital for one not to be infected?’ (Pt19, 30 years, black person)

Some of the women who inject Nyaope in this study indicated that they participate in sex work or transactional sex to raise money for drugs. The relationship between multiple sex partners, sex work and HIV is well documented, PrEP will curb HIV amongst women who inject Nyaope.

### Human immunodeficiency virus counselling and testing

Participants shared that making HIV counselling and testing (HCT) available to women who inject Nyaope can curb HIV incidence amongst women who inject Nyaope. The HCT should be conducted as an outreach, instead of participants going to them, the HCT service providers should target drug hotspots where PWID gather to test them. This is supported by the following participant:

‘I think by going out to the community doing all this (testing), like open the test (centres) where everyone is free; like where they smoke (drug hotspot), like smoking zone or something like that, maybe to go there and to offer them for HIV testing.’ (Pt13, 28 years, mixed race person)

## Economic intervention

### Employment opportunities

Participants are of the view that employment will influence their chances of participating in sex, which increases the likelihood to contract HIV infection, and they will further stop to depend on other people. This is supported by the following narrative statements:

‘As females, we are unemployed, if we can get the opportunity to work for ourselves, I believe HIV among females who inject Nyaope will decrease, because some of us when we are broke we depend on other users. For example, someone can come without an injection and ask you for your injection; knowing that she or he will leave something for you, you will give them an injection. At that point, I do not know if the person is HIV infected or not. That is how we get infected.’ (Pt3, 34 years, black person)

Some women who inject Nyaope are pressured to ‘sell sex’ to finance their drug use. Unemployment increases the risk of contracting HIV infection through unprotected sex and engaging in the sharing of needles:

‘We should be hired for part time jobs. It is painful to stay the whole day without using. Let them work for it because they understand how difficult it is to stay without it the whole day and not smoke, let them given part time jobs.’ (Pt9, 31 years, black person)

‘We should get part time jobs, it is painful to think that, if now you want R100, you need to sleep with five people. But if one has a part time job that would give them R100 in a day it would be much better.’ (Pt5, 31 years, black person)

Some participants emphasised the pain of not having an income to maintain using drugs. The withdrawals influence them to engage in practices which they would not engage in if they did not need money for drugs. Participants are of the view that if they can find work, working will interrupt their routine of using drugs, which will decrease the risk of contracting HIV and other blood-borne infections.

### Educational intervention

Some participants reported that support groups and awareness campaigns are key interventions that can assist in empowering them to make informed decisions, which will protect them against HIV infection.

### Support groups

Some participants acknowledge that support groups will empower them to engage in safe injecting and other behaviours, which will reduce their risk of contracting HIV. They further emphasised that the support groups must be conducted within communities. This means they must be accessible to all the female Nyaope injectors.

Participants reported that:

‘There must be programmes, were females will come together and be taught about the dangers of using drugs, sexual abstinence, and encourage those who are HIV positive to live a healthy life.’ (Pt10, 32 years, black person)

‘Get the ladies together and let us talk and listen to each other.’ (Pt14, 23 years, black person)

‘Support groups so that they can lean more because other people do not really understand what HIV is. I think through support groups, which must be within the community, we will learn.’ (Pt13, 28 years, mixed race person)

‘They can have lessons every month, which will remind them now and then about safe injecting.’ (Pt8, 35 years, black person)

‘Because of Nyaope we go through a lot of things I never thought I would do in my life, so if we can come together.’ (Pt1, 23 years, black person)

The support groups may focus on teaching safe injecting techniques, overdose prevention, safe sex, healthy living and provide support for group members. The support group can act as resources for group members and safe space to establish friendships. For those who desire to stop using drugs can also benefit from the support group. During the support groups, other service providers can bring their services like Reproductive Health Services and sterile needles:

‘More support groups should be provided and maybe contraceptives and condoms as well.’ (Pt4, 22 years, black person)

### Awareness campaigns

Participants alluded that hope is not lost, and efforts must be strengthened to curb HIV incidence amongst women who inject Nyaope. Participants indicated that awareness campaigns should be conducted and they should go to the hotspots. They should go where female Nyaope injectors meet to use their drugs. They further indicated that as campaigns are presented, service providers should take injections with them to give females who are injecting Nyaope. The awareness should use all sorts of platforms; including television. Television programmes can target audiences. They can target people who are injecting and teach them about safe injecting, and help the community understand the complexities of injecting, and aim at reducing the stigmatisation against female Nyaope injectors:

‘Spread the information, informing women who inject Nyaope not to share needles, it is important that they know, HIV is not about having sex, but one can get it through sharing used needles. People who share the information should, go to places where there are smokers (drug hotspots).’ (Pt7, 24 years, black person)

‘Go to the people who are smoking and educate them about possibilities of contacting infections when sharing needles.’ (Pt23, 31 years, black person)

‘There should be campaigns to the hot spots where they meet to smoke. Maybe the campaigns must be once or twice in a week and give people who inject the injections.’ (Pt9, 31 years, black person)

‘Even on the television, if they can make a programme or something or documentary to show the risk of woman to be easily infected from HIV when coming to using injections.’ (Pt18, 29 years, black person)

## Discussion

The results of this empirical study indicate that HIV amongst women who inject Nyaope can be curbed by multiple interventions. The findings highlight health, economic and educational intervention can be utilised to curb the HIV incident amongst women who inject Nyaope.

Programmes that offer disinfected injecting equipment are highly effective in decreasing transmissions of HIV and other blood-borne infections.^21^ Needle Exchange Programme has a lot of potential to address needle sharing amongst women who inject Nyaope when implemented. The needle exchange programme goes without criticism. Participants indicate that NSP should offer them enough needles, it should go where users are at and it should not run out of stock. Fear of leaving users without needles was recorded as the participant indicated that in the case of NSP not been able to give them needles, women who inject Nyaope will resort to needle sharing. It is therefore imperative that the NSP programme should manage its capacity to always deliver services to PWID.

The needle exchange programme has been shown to decrease risky behaviour and to provide a critical link to care for PWID, who may receive all or most of their services through their NSP.^22^ These valuable benefits occur without any known downsides, as NSPs often reduce syringe waste found in the community, decrease needle stick injuries, and have no negative impacts on crime or amount of drug use. Needle exchange programme comes with controversy which has mainly been fuelled by the notion of whether a person who uses drugs should be seen as a criminal or a patient, and whether it is ethical or lawful to provide a person using drugs the means to inject. This assumption is usually the held by opponents of NSPs who greatly support drug treatment programmes that are based on abstinence and ‘drug-free’ treatment for addiction. Needle exchange programmes have demonstrated to be a cost-effective way of curbing HIV in other low-income settings.^23^

Women who inject Nyaope are more probable to practice transactional sex and sex work.^24^ Distribution of condoms and educating women who use Nyaope on condom use will empower the women to use condoms consistently to protect themselves and their sex partners against HIV infections. Female Nyaope injectors are more probable to practice high sexual risk as a result of the severe discrimination and cultural stigmatisation attached to injecting drug use.^12^

Irrespective of a person’s knowledge of HIV status or ART compliance level, it is projected that the accurate and constant use of condoms decreases the risk of sexual transmission of HIV infection by 70% in heterosexual couples. Condom supply interventions have, for many years, been a backbone of public health HIV prevention efforts.^25^

The participants highlighted that the introduction and accessibility of PrEP to female Nyaope injectors will assist in curbing HIV incidence amongst women who inject Nyaope. Pre-exposure prophylaxis is essential for female Nyaope injectors because it can empower them to protect themselves from HIV infection.^26^ More educational programmes targeting women who inject Nyaope ought to be conducted to introduce these prevention treatments. Pre-exposure prophylaxis has conceivable worth in the social and sexual networks of PWID where the prevalence of HIV is high. Pre-exposure prophylaxis will escalate its HIV prevention prospects when it is introduced in a way that supplements and reinforces existing harm reduction and health promotion activities.^27^

The price of PrEP is substantially lesser than offering antiretroviral therapy to an infected person for the period of their lifetime. In countries where HIV PrEP is available, including SA, awareness of and access to it is low amongst female injectors.^28^ Pre-exposure prophylaxis is predominantly vital for women who inject nyaope because it can empower them to protect themselves from HIV infection.^26^ Pre-exposure prophylaxis has conceivable worth in the social and sexual networks of PWID where the prevalence of HIV is high. Pre-exposure prophylaxis will appreciate its HIV prevention prospects when it is introduced in a way that supplements and reinforces existing harm reduction and health promotion activities.^27^

Human Immunodeficiency Virus Counselling and Testing should not be neglected as part of strategies aimed at curbing HIV amongst WWID. Female Nyaope injectors are active members of the community. They are active in transmitting and also contracting HIV. Overlooking them might make the 90–90–90 plan only a dream, which will never be realised. Instantaneous HIV diagnosis, proper link to care, early treatment, retention in care and sustained viral suppression are all important in reducing morbidity and mortality from HIV.^29^ More efforts should be put to scale up at getting women who inject Nyaope tested for HIV. Instead of waiting for the women who come to where testing is done, HIV testing services must go to where people are.^30^ Human Immunodeficiency Virus Counselling and Testing is the first step for multiple interventions for HIV prevention and care, and further serves as an entry point for HIV prevention services.^31^

Poor socioeconomic status can act as an obstacle to educational prospects, the opportunity to approach healthcare and employment, generating a conducive environment for HIV incidence.^32^ Participants indicated that they are unemployed and employment opportunities will assist them to take care of themselves instead of depending on their people. The participants participate in sex work to raise money, which is not something they are happy with, but they are compelled by circumstances they find themselves in. Poor prospects of unemployment add to the dual risk of contracting HIV infection, through unprotected sex and engaging in sharing needles.^8^

The establishment of locally based support groups was indicated to have the potential to curb HIV amongst women who inject Nyaope. Forming peer support groups of female Nyaope injectors is critical to neutralise the self-stigma. It can also reinforce females’ communication skills which assist them in their relationships. This will help them to access social and health services.^29^ Such groups may serve the purpose of sharing experiences, encouraging disclosure, reducing stigma and discrimination, improving self-esteem, enhancing women’s coping skills and psychosocial functioning, and supporting medication adherence and improved retention in HIV care.^33^ Support groups for WWUD were also brought forward by participants in the UNODC^15^ study, with an excess importance placed on the importance of these groups being for WWUD. The environment of these support groups should promote relationship-building amongst the participants, as well as provide tools on how best to better one’s view of self and one’s surroundings.^15^ By joining support groups, people appreciate that they are not alone in their condition.^33,34^

The participants reported that hope is not lost and efforts must be strengthened to curb HIV incidence. The participants indicated that awareness campaigns should be conducted and they should go to the drug hotspots. They should go where female Nyaope injectors meet to use their drugs. They further indicated that as campaigns are presented, service providers should take injections with them to give females who are injecting Nyaope. The awareness should use all sorts of platforms; including television. Television programmes can target audiences. They can target people who are injecting and teach them about safe injecting and help the community understand the complexities of injecting and aim at reducing the stigmatisation against female Nyaope injectors. The awareness can go beyond drug users and educate the community and local leadership to understand that drug use is an indication of social glitches that confront female Nyaope injectors, rather than an issue of simple individual choice that abstinence and prohibition can solve.^29^

## Limitations and strengths

The limitation of the study are; the study considered only participants who were accessing a service at selected COSUP sites. The research employed a qualitative research design, the findings cannot be generalised to all women who inject Nyaope. Few studies were conducted on PWID within the South African context; only one study on female Nyaope injectors in SA was conducted by UNODC in 2017. The researcher depended on international research for literature. The sample consisted of WWID regardless of whether they stay with family or are homeless, employed or unemployed.

The strength of the study is, this research focused on women who are injecting Nyaope. Women who inject Nyaope are often overlooked, and they were difficult to recruit to participate in the study. The sample consist of 24 women participants.

## Recommendations

The findings of the study demonstrate that HIV incidence amongst women who inject Nyaope can be curbed or mitigated. It further identifies harm reduction programmes as having the potential to address the risky behaviours of WWID. SA Government should take a leading role in implementing harm reduction as an SUD strategy to curb HIV incidence amongst WWID, and to all people who use and inject drugs. More studies on PWID should be conducted to understand the South African drug injecting context better, instead of understanding South African drug injecting context using international research.

## Conclusion

Nyaope injecting has brought the field of SUD treatment and HIV prevention together. Those who study HIV prevention cannot do so without taking into consideration women who inject Nyaope. Furthermore, those who provide SUD treatment cannot do so without embracing the continuous risk of HIV infection confronting women who inject Nyaope daily.

Lack of needles increases the likelihood to share needles and spread HIV and other blood-borne infections amongst the drug injecting social network. Participants have reported health intervention, economic intervention and educational interventions as key to curbing HIV incidence amongst women who inject Nyaope. Most of the reported interventions exist in SA, but what is needed is good coordination to include women who use nyaope as beneficiaries.

Nyaope injecting practice is multifaceted, it requires a multidisciplinary approach. The findings of the study pleas for implementation of a harm reduction programme, which is cost-effective, and beneficial for the health outcomes of people who use drugs, their families and communities.^14^ Department of Health and Department of Social Development, should jointly adopt a primary role in addressing Nyaope injecting practice as partners.

The findings of this study may also be relevant and applicable to women who inject other drugs, for example, cocaine, methamphetamines, ecstasy, ketamine amongst others. Mechanisms to curb HIV amongst women who inject Nyaope exist, and when implemented, have the potential to address high HIV incidence amongst women who inject Nyaope
